# A Significant Fluorescence Turn-On Probe for the Recognition of Al^3+^ and Its Application

**DOI:** 10.3390/molecules27082569

**Published:** 2022-04-15

**Authors:** Zhiyong Xing, Junli Wang, Junhui Huang, Xiangfeng Chen, Ziao Zong, Chuanbin Fan, Guimei Huang

**Affiliations:** 1 School of Laboratory Medicine, Youjiang Medical University for Nationalities, Baise 533000, China; 01327@ymun.edu.cn (X.C.); zongziao@ymun.edu.cn (Z.Z.); 01363@ymun.edu.cn (C.F.); 00966@ymun.edu.cn (G.H.); 2 Department of Reproductive Medicine, Affiliated Hospital of Youjiang Medical University for Nationalities, Baise 533000, China; 3 Environmental Health Risk Assessment and Prevention Engineering Center of Ecological Aluminum Industry Base, Youjiang Medical University for Nationalities, Baise 533000, China; 4 Institute of Science and Technology Information, Baise 533000, China; 00497@ymun.edu.cn

**Keywords:** benzothiazole, fluorescence, Al^3+^, cell image

## Abstract

An easy prepared probe, **BHMMP**, was designed and synthesized, which displayed a significant fluorescence enhancement (over 38-fold) and obvious color change in the recognition of Al^3+^. The binding ratio of probe **BHMMP** to Al^3+^ was determined as 1:1, according to Job plot. The binding mechanism was fully clarified by the experiments, such as FT-IR spectrum, ESI–MS analysis, and ^1^H NMR titration. A DFT study further confirmed the binding mode of **BHMMP** to Al^3+^. The limit of detection (LOD) for Al^3+^ was determined as low as 0.70 µM, based on the fluorescence titration of **BHMMP**. Moreover, the results from real sample experiments, including real water samples, test papers, and cell images, well-demonstrated that **BHMMP** was capable of sensing Al^3+^ in environmental and biological systems.

## 1. Introduction

As is known to us, aluminum exists widely in the earth and keeps in close touch with our daily life, such as packaging materials, electrical devices, kitchenware, and pharmaceutical synthesis [1,2,3,4]. Nevertheless, ingested aluminum ions can accumulate in different organs and cause significant toxicity to damage creatures [5,6]. Some researchers have indicated that high levels of aluminum ions in soil and water resources can impede plant growth and severely influence marine life [7,8,9]. In addition, it also can damage the human’s nervous system and immune system, while its accumulation exceeds the tolerable level in human body, thus leading to serious diseases, such as Alzheimer’s disease and dialysis dementia syndrome [10,11,12,13]. Therefore, it is essential to detect Al3+ by qualitative and quantitative analyses for further environmental protection and biological health maintenance.

There are many analytical methods available for monitoring metal ions, including atomic absorption spectrometry [14,15], inductively coupled plasma mass spectrometry [16], and anodic stripping voltammetry [17]. Compared with the above detection methods, fluorescence analysis has gradually become an effective tool in the field of analysis and detection, not only due to its advantages of high sensitivity, as well as low detection limit, but also owing to its visual recognition and low intracellular toxicity [18,19,20,21,22,23,24,25,26,27,28,29]. Considering some Al^3+^ fluorescent probes suffer from unacceptable factors, such as the complex synthesis routes, interferences by other co-existence metal ions, and insolubility in water, the development of an efficient Al^3+^ fluorescent probe with high sensitivity, as well as good application prospects, still attracts worldwide attention [2,30,31,32,33,34,35].

**Scheme 1:**
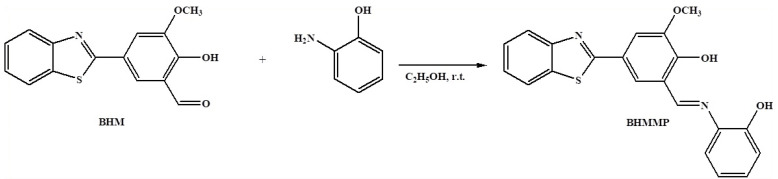
Synthesis procedure of the probe **BHMMP**.

Benzothiazole is an excellent fluorophore in design fluorescent probes, as it has good photostability and high fluorescence quantum yield [36,37,38,39]. Meanwhile, Schiff base ligands are coordinated to specific metal ions, which leads to the prevention of C=N isomerization, thereby enhancing fluorescence [40,41,42]. So, the introduction of a special sp2-hybridized nitrogen (CH=N) functional group was used to compensate for the lack of spectral characteristics, inadequate coordination, and strong hydration ability of aluminum ions [43,44]. Enlightened by our previous work, a novel Schiff base fluorescent probe (*E*)-4-(benzo[d]thiazol-2-yl)-2-(((2-hydroxyphenyl)imino)methyl)-6-methoxyphenol (**BHMMP**) was easily synthesized and systematically investigated (Scheme 1), presenting a turn-on fluorescent response towards Al^3+^, which was ascribed to the inhibition of C=N isomerization and photo-induced electron transfer (PET) processes. A comparison of the probe **BHMMP** with the ones with the similar group was provided in Table S1 [45,46,47,48,49,50,51]. The unique advantages of **BHMMP** showed high sensitivity, good water solubility, significant recognition signal, and excellent potential application capabilities.

## 2. Results and Discussion

### 2.1. Fluorescence and UV–Vis Spectral Response of BHMMP to Al^3+^

**Figure 1:**
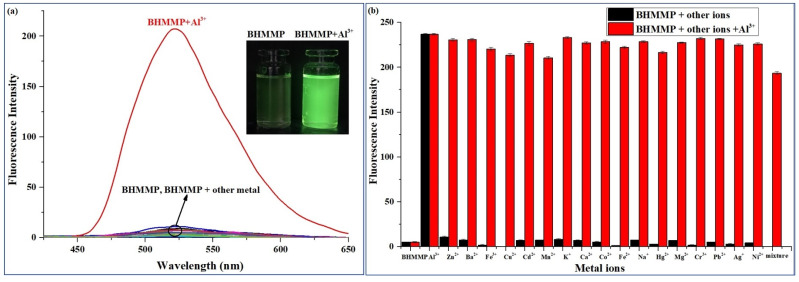
(**a**) Fluorescence spectrum changes of **BHMMP** (10 μM) in EtOH/H_2_O (2/3, *v*/*v*, 0.01 M HEPES, pH = 5) after adding different metal ions (50 μM), λ_ex_ = 370 nm. Inset: visual fluorescence change of **BHMMP** solution upon addition of Al^3+^ under UV illumination at 365 nm. (**b**) The emission intensity of **BHMMP** (10 μM) solution containing Al^3+^ (50 μM), as well as the same amount of interfering ions at 522 nm.

Firstly, one of the most essential characteristics of fluorescent probe is its excellent selectivity; so, a fluorescence selectivity experiment was conducted on the fluorescence emission spectrum of the solution of probe **BHMMP** (10 μM) with different metal ions (50 μM) (including Al^3+^, K^+^, Mg^2+^, Mn^2+^, Na^+^, Ni^2+^, Ag^+^, Ca^2+^, Cd^2+^, Co^2+^, Cr^3+^, Cu^2+^, Fe^2+^, Pb^2+^, Zn^2+^, Hg^2+^, and Fe^3+^) in EtOH/H_2_O (2/3, *v*/*v*, 0.01 M HEPES, pH = 5) at room temperature. As depicted in Figure 1a, there was a weak fluorescence emission for **BHMMP** alone, with a fluorescence quantum yield (Φ_f_, quinine sulfate as standard) as low as 0.005 [52]. In the case of the existence of Al^3+^ in **BHMMP** solution, the fluorescence spectrum displayed a significant enhancement with the maximum emission peak at 522 nm (Φ_f_ = 0.11), alongside fluorescence color, which was changed from colorless to yellow-green under ultraviolet light. It was conjectured that the increase of fluorescence might be associated with the coordination of **BHMMP** to Al^3+^, resulting in the enhancement of structure rigidity of the probe, which could prevent PET and C=N isomerization processes [53,54]. Under the same conditions, other metal ions did not cause significant changes in fluorescence intensity, indicating that the probe showed a highly sensitive “turn-on” fluorescence sensing behavior in the presence of Al^3+^. Competition experiments were measured to validate the fluorescence sensing property of **BHMMP** to Al^3+^. Adding the same amount of other metal cations and the mixture (all tested ions) into the **BHMMP** solution containing Al^3+^ (5 eq.), the fluorescence intensity at 522 nm had almost no obvious changes (Figure 1b), which confirmed that **BHMMP** was more intimate with Al^3+^ in the presence of other ions and could be viewed as a selective fluorescent probe for measuring Al^3+^ in complex environments.

**Figure 2:**
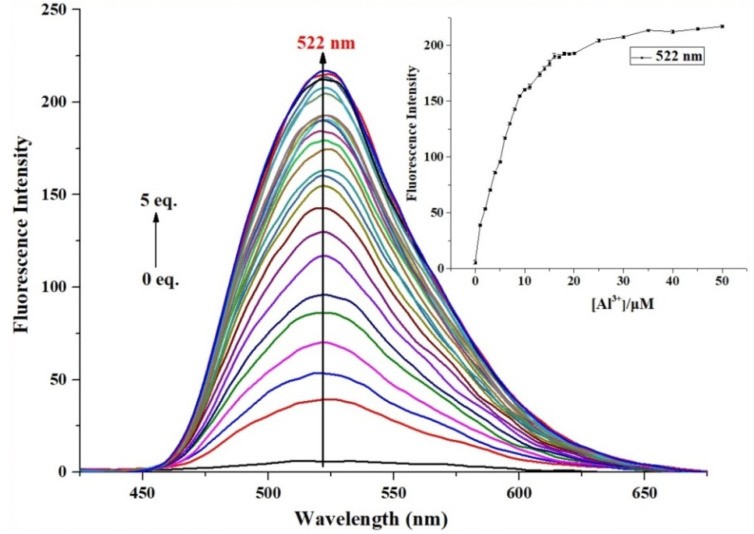
Fluorescence titration spectrum of **BHMMP** (10 µM), with varying ratios of Al^3+^ (0–5 eq.) in EtOH/H_2_O (2/3, *v*/*v*, 0.01 M HEPES, pH = 5) medium, λ_ex_ = 370 nm. Inset: The variation trend of fluorescence intensity of **BHMMP** at 522 nm with increasing Al^3+^ concentration.

Fluorescence titration experiments were performed to study the quantitative fluorescence sensing ability of **BHMMP** to Al^3+^. During the process of titration, a fluorescence emission peak at 522 nm raised gradually with the increase of Al^3+^ concentration, and the fluorescence intensity stabilized a maximum value in the presence of two equiv. of Al^3+^ (Figure 2). According to the titration data mentioned above, it was found that the emission intensity of **BHMMP** was linearly related to the Al^3+^ concentration, in the range of 1.0–10.0 μM (R^2^ = 0.9908), which indicated that **BHMMP** could be successfully used as a quantitative tool to evaluate Al^3+^ (Figure S1). The detection limit (LOD) was obtained from the result of fluorescence titration as 7.04 × 10^−7^ M (3σ/S, where σ is the standard deviation of the blank solution, and S is slope from plotting the fluorescence intensity versus the concentration of Al^3+^), which was below the guideline value of Al^3+^ in drinking water prescribed by WHO (the maximum concentration was 7.4 μM) [55,56].

**Figure 3:**
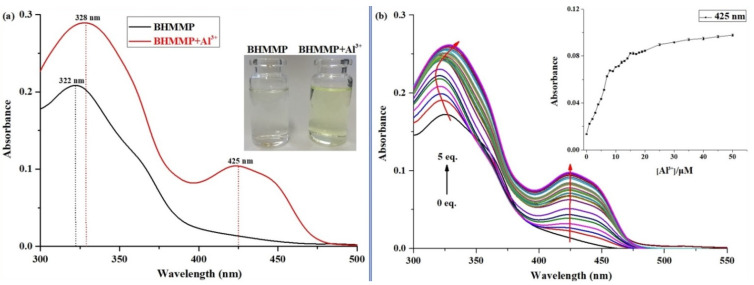
(**a**) UV–Vis absorption spectral of **BHMMP** (10 µM), in the absence and presence of Al^3+^ (5 eq.). Inset: color change of **BHMMP** solution before and after addition of Al^3+^ in ambient light. (**b**) Absorption titration spectra of **BHMMP** (10 µM) with increasing Al^3+^ concentration (0–50 µM). Inset: the variation trend of absorbance of **BHMMP** at 425 nm, upon the gradual addition of Al^3+^.

In the UV spectrum experiment (Figure 3a) showed that, after adding five equiv. of Al^3+^ to the **BHMMP** solution, a slight red-shift of the absorbance peak from 322 to 328 nm, along with the appearance of a new peak at 425 nm, was observed, which suggested the stable complex formation between **BHMMP** and Al^3+^. At the same time, the presence of Al^3+^ resulted in the increase of the conjugated degree, thus causing the solution to alter obviously from colorless to pale yellow. Furthermore, it was worthwhile to mention that the absorption value of **BHMMP**–Al^3+^ at 425 nm was positively correlated with the concentration of Al^3+^, from which the LOD was calculated to be 4.18 × 10^−7^ M (Figure 3b). The above results confirmed that the potential application of **BHMMP**, in the quantitative determination of Al^3+^, was expected in analytical chemistry and biological systems.

### 2.2. Binding Mode Studied

**Figure 4:**
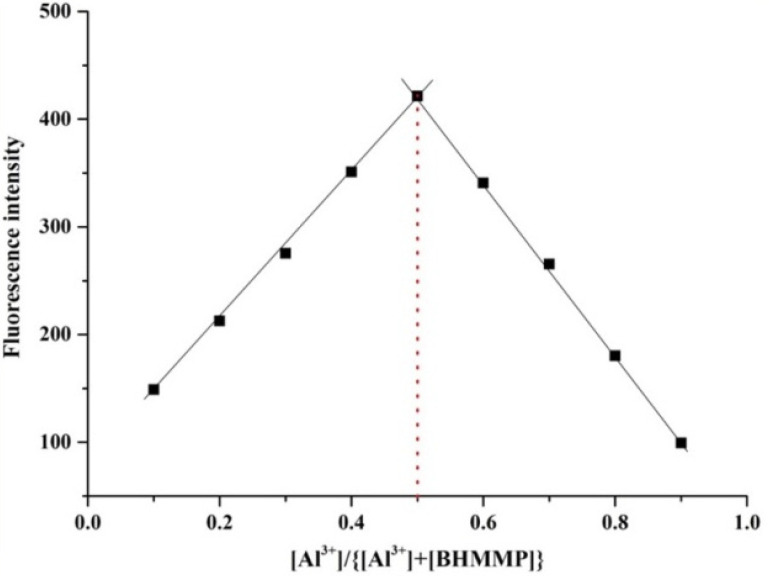
Job plot for evaluating the stoichiometry of **BHMMP** and Al^3+^ with fluorescence spectra (λ_ex_=370 nm, λ_em_=522 nm).

**Figure 5:**
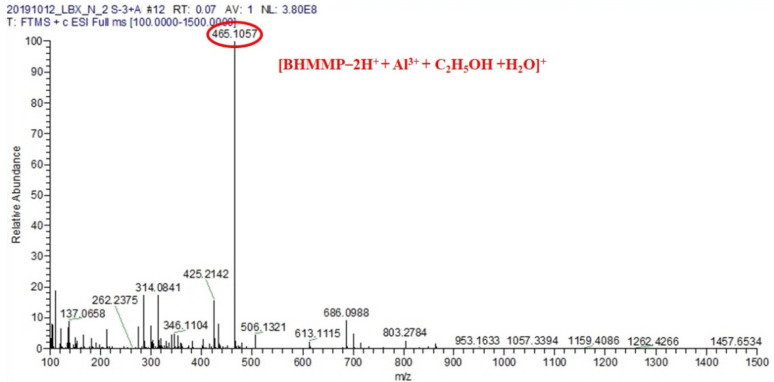
The ESI–MS spectrum of **BHMMP**–Al^3+^ complex in positive ion source mode.

The Job plot method was adopted to infer the stoichiometric relationship between **BHMMP** and Al^3+^ (Figure 4). To this end, nine groups of solutions, with continuously varying mole fraction of guest [Al^3+^]/([**BHMMP**+Al^3+^]), were prepared by maintaining the total concentration of the mixed system at 5 × 10^−5^ M. The emission intensity reached the maximum when the abscissa of the Job curve was 0.5, indicating the coordination ratio of **BHMMP**–Al^3+^ complex was 1:1 in EtOH/H_2_O (2/3, *v*/*v*, 0.01 M HEPES, pH = 5), which was further supported by mass spectroscopy analysis, as follows. A mass spectra signal at 465.1056 *m*/*z* was attributed to [**BHMMP**+Al^3+^+C_2_H_5_OH+H_2_O-2H^−^]^+^ (calcd: 465.1065), which proved that the stoichiometry of the chelate is 1:1 and revealed that the coordination sphere of Al^3+^ was composed of solution components, such as water and ethanol (Figure 5). There was a strong binding affinity of **BHMMP** with Al^3+^, and the association constants calculated from the results of fluorescence and absorption titration experiments were 3.10 × 10^4^ (Figure S2) and 2.55 × 10^3^ M^−1^ (Figure S3), by the Benesi–Hildebrand plot, respectively [57,58,59].

**Figure 6:**
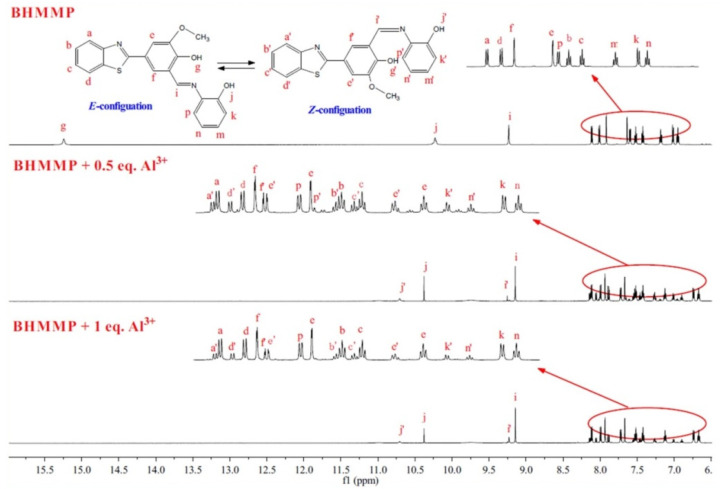
^1^H NMR spectra of **BHMMP** in the presence of different equivalents of Al^3+^ in DMSO-*d*_6_.

**Figure 7:**
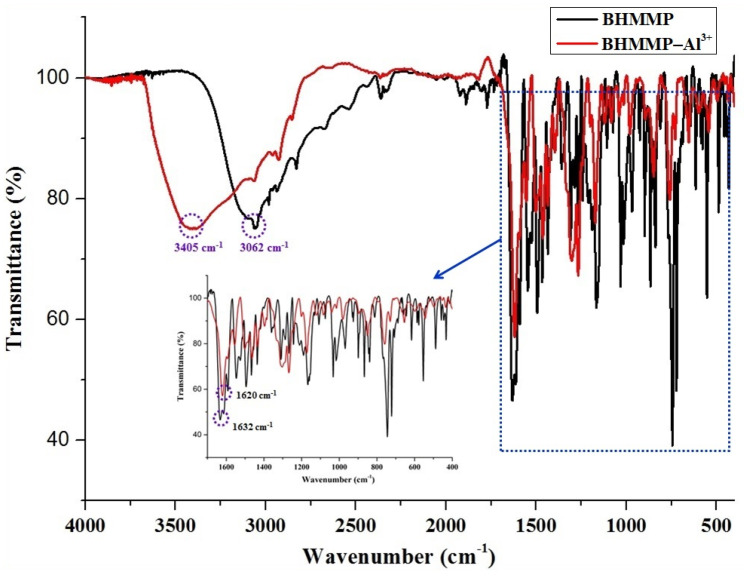
The FT-IR spectrum of **BHMMP**, compared to the **BHMMP**–Al^3+^ complex.

To clarify detailed information about the interaction mode between **BHMMP** and Al^3+^, the ^1^H NMR and FT-IR spectrum of **BHMMP** were recorded. The result of ^1^H NMR titration experiment was obtained by adding different equivalent Al^3+^ to several **BHMMP**, as illustrated in Figure 6. By comparison, the resonance signal of phenolic hydroxyl group (Hg), at around 15.24 ppm, was completely curtailed, supporting the occurrence of deprotonation upon the combination of **BHMMP** with Al^3+^. The peaks at 10.23 and 9.23 ppm, belonging to the hydroxyl (Hj) and imine (Hi) groups, were gradually shortened; meanwhile, their corresponding signals were appeared at 10.71 (Hj’) and 9.25 ppm (Hi’), due to the transition of (*Z*)-configuration to (*E*)-configuration, caused by the formation of complex. In addition, the chemical shifts of protons in the aromatic ring (from 6.94 ppm to 8.12 ppm) showed obvious changes, which provided strong evidence for the existence of the two spatial configurations. These observations indicated that nitrogen atom on imine and oxygen atoms on two phenolic hydroxyl groups participated, in coordination with Al^3+^. As for the FT-IR spectra of **BHMMP** and **BHMMP**–Al^3+^ complex, the stretching vibration peak, attributed to C=N, was shifted from 1634 to 1620 cm^−1^, which was consistent with the conclusion that imine was taking part in the complexation process (Figure 7).

### 2.3. DFT Study

**Figure 8:**
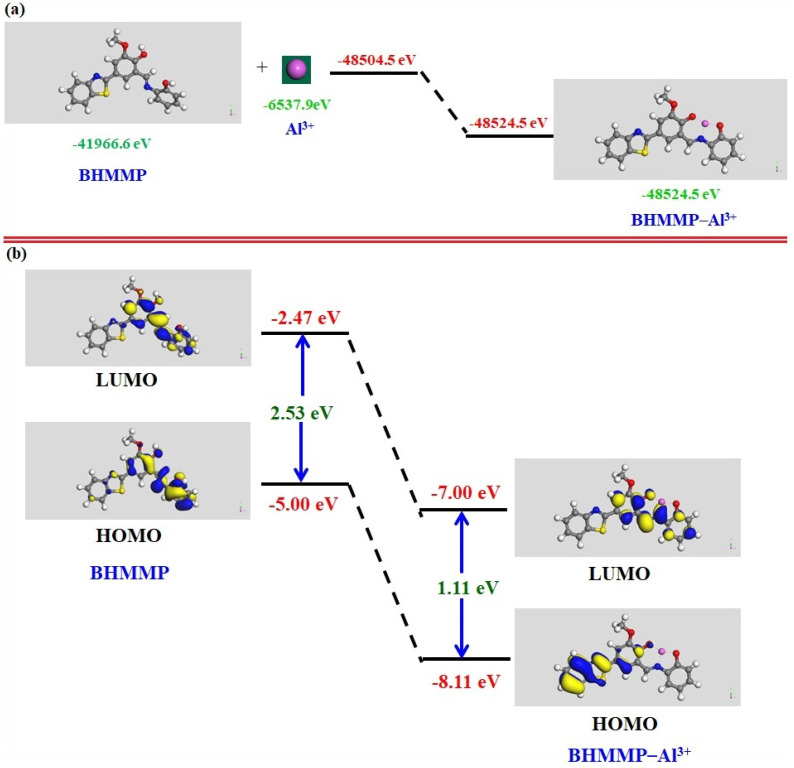
(**a**) Energy optimized structure of probe **BHMMP** (left) and (BHMMP–Al^3+^) (right), and (**b**) the corresponding molecular orbital of **BHMMP** and **BHMMP**–Al^3+^.

The comparison of total energy between **BHMMP** (**BHMMP** = −41,966.6 eV) and the **BHMMP**–Al^3+^ complex (**BHMMP**–Al^3+^ = −48,524.5 eV) indicated that the **BHMMP**–Al^3+^ complex was highly stable (Figure 8a). Moreover, the energy of either the highest occupied molecular orbital (HOMO) or the lowest unoccupied molecular orbital (LUMO) of the **BHMMP**–Al^3+^ complex was significantly lower than that of free **BHMMP** (Figure 8b), and the energy gap of HOMO-LUMO of **BHMMP**–Al^3+^ (calculated as 1.11 eV) was decreased in comparison with that of **BHMMP** (calculated as 2.53 eV), which indicated that the complex formation of **BHMMP** and Al^3+^ was more stable than **BHMMP**. Meanwhile, Tauc plots (Figure S4) were illustrated, according to the absorption spectra data of **BHMMP** and **BHMMP**–Al^3+^, and the optical energy gap of **BHMMP**–Al^3+^ (estimated as 2.5 eV) also decreased, in comparison with that of **BHMMP** (estimated as 3.1 eV). Based on the above analysis, the experimental and theoretical results were consistent with the conclusion that the energy gap of **BHMMP** will decrease after the addition of Al^3+^.

**Scheme 2:**
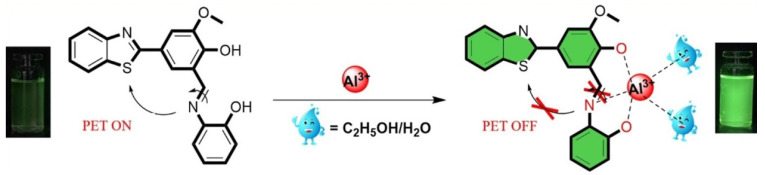
Proposed recognition mechanism for the fluorescence enhancement of **BHMMP** to Al^3+^ and possible structure of **BHMMP**–Al^3+^ complex.

Combining the above fluorescence and IR spectroscopy, HRMS and ^1^H NMR titration, and DFT study, the possible structure of **BHMMP**–Al^3+^ was reasonably speculated (Scheme 2), and the mechanism of fluorescent enhancement was attributed to the hindrance of the PET and C=N isomerization processes by the establishment of the complex.

### 2.4. Effect of pH and Response Time Study

**Figure 9:**
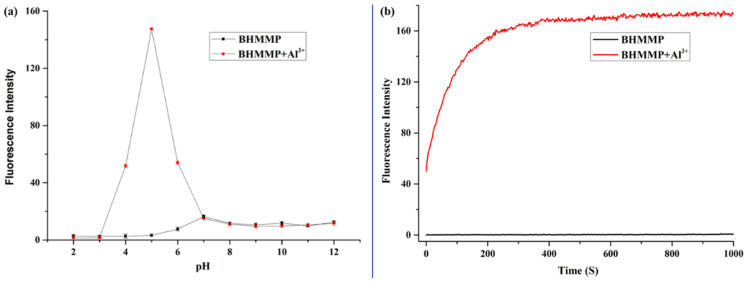
(**a**) Effect of pH on the fluorescence intensity of **BHMMP** and the **BHMMP**–Al^3+^ complex, λ_ex_ = 370 nm. (**b**) Time-dependent fluorescence intensity changes of **BHMMP** (10 μM) to Al^3+^ (50 μM) at 522 nm.

Considering that the probe is usually affected by the proton concentration in the medium during the recognition of ions, the effect of pH on the fluorescence spectrum of BHMMP in the absence and presence of Al^3+^ was explored at different pH values (Figure 9a). The fluorescence intensity of free probe **BHMMP** at 522 nm was weak, in a pH range from 2 to 12, indicating that the probe was insensitive to H^+^/OH^−^. However, a significant increase in the emission intensity of **BHMMP** between pH 4 and 6 was observed, so the probe was suitable for detecting Al^3+^ in faintly acidic medium.

The response time enabled us to reflect the sensitivity and stability of the probe. The changing law of the fluorescence intensity with time was monitored in medium EtOH/H_2_O (*v*/*v*, 2/3, 0.01 M HEPES, pH = 5) (Figure 9b). After the addition of Al^3+^, the fluorescence signal of BHMMP responded instantaneously, reaching the maximum at 3 min, and maintained constant at more than a quarter of an hour. The result sufficiently confirmed that the complexing process between **BHMMP** and Al^3+^ was rapid and stable.

### 2.5. Reversibility Study

The reversibility of **BHMMP** (10 µM) was investigated in the EtOH/H_2_O (*v*/*v*, 2/3, 0.01 M HEPES, pH = 5) solution by adding EDTA, which was a good chelating agent with Al^3+^ (Figure S5). Upon the addition of Al^3+^ (50 µM) into the solution of **BHMMP**, the fluorescence spectrum had significant changes, compared with the correspondence spectrum of **BHMMP** itself. However, after the addition of EDTA (50 µM) to the solutions of **BHMMP**–Al^3+^, the fluorescence spectra of the solution of **BHMMP**–Al^3+^ showed much more similar correspondence to that of **BHMMP** in the absence of Al^3+^, indicating the recovering of the **BHMMP** (Figure S5a). This result was also supported by the bonding constant of **BHMMP** with Al^3+^, calculated as 3.10 × 10^4^, which was far lower than that of EDTA, with Al^3+^ calculated as 1.99 × 10^16^ M^−1^ [60]. However, the alternate addition of Al^3+^ (50 µM) and EDTA (50 µM) to the above-mentioned solution, the fluorescence intensity at 522 nm was almost constant with that of **BHMMP** itself (Figure S5b). This result indicated that **BHMMP** was a non-reusable probe in sensing Al^3+^.

## 3. Practical Applications

### 3.1. Quantitative Detection of Al^3+^ in Actual Water Samples

To measure the feasibility of probe **BHMMP** to quantitatively detect Al^3+^ in real aqueous sample, tap water and Songhua River water samples were spiked with different concentrations of Al^3+^ solution and analyzed by the proposed fluorimetric method (Figure S6). The fluorescence intensity in the above experiment was collected, and the recoveries were within the range of 97–102%, indicating that the probe had the potential capacity to conduct trace analysis on Al^3+^ in environmental water samples (Table S2).

### 3.2. Monitoring Al^3+^ on Test Paper

**Figure 10:**
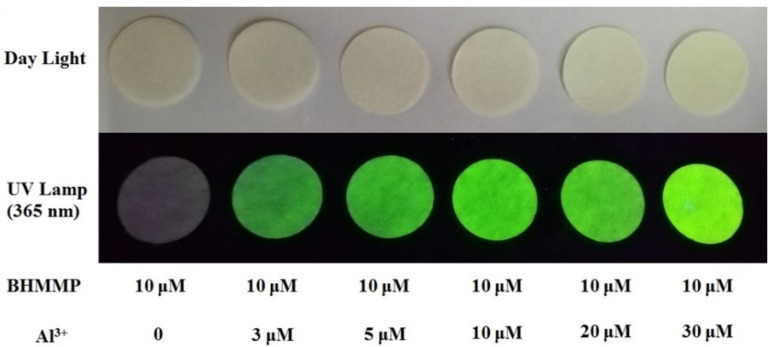
Photographic image of different concentrations of Al^3+^ by **BHMMP** (10 μM) on test strip by daylight and UV light.

The color changes of the mixed solution containing different concentrations of Al^3+^ were observed clearly under sunlight and 365 nm ultraviolet light (Figure 10). Enlightened by this, we conducted colorimetric experiments on Al^3+^ by loading **BHMMP** on the test paper [61,62]. The test paper was soaped into **BHMMP** solutions, with various amounts of Al^3+^, and then dried naturally. As the concentration of Al^3+^ increased, the color of the test strip changed from colorless to yellow-green under 365 nm UV light, which indicated that **BHMMP** was expected to become a portable tool for detecting Al^3+^.

### 3.3. Cellular Imaging Experiments

**Figure 11:**
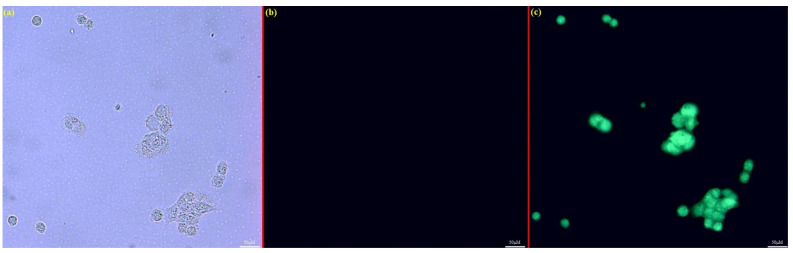
Images of HSC cells: cells themselves, (**a**) bright field, only incubated with 10 μM **BHMMP**; (**b**) fluorescence treated with 10 μM **BHMMP,** followed by addition of 50 μM Al^3+^; (**c**) fluorescence.

Due to the serious toxicity of aluminum ions on living organisms, successful imaging in biological systems is an essential practical application capability for excellent Al^3+^ probes. Fluorescence images of human stromal cells (HSC) treated with Al^3+^ were acquired by a fluorescence microscopy [63,64]. As depicted in Figure 11, it could be observed that either the cells themselves (Figure 11A), or incubated with **BHMMP** (Figure 11B), did not all emit fluorescence; however, the unique light-green fluorescence in cells cultured with **BHMMP** (10 μM) and Al^3+^ (50 μM) was monitored, which might be the coordination of **BHMMP** and Al^3+^ inside the cells (Figure 11C). Hence, the probe **BHMMP** was still capable of sensing Al^3+^ in cells.

## 4. Materials and Methods

### 4.1. Materials and Instruments

Materials (such as vanillin, 2-aminophenol and 2-aminothiophenol) for synthesis and metal salts were obtained from Energy Chemical (Shanghai, China). The required solvents and reagents were of analytical or spectroscopic reagent grades during the overall experiments. The structure of the target compound and its coordination mode with metal ions were studied on the Bruck AV-600 MHz system, Waters Xevo UPLC/G2-SQ Tof MS, and Bruker ALPHA-T spectrometers. The spectral properties of the compound are explored, in detail, through a Shimadzu UV-2700 UV–vis spectrometer and Perkin Elmer LS55 fluorescence spectrometer. Test solutions of different pH values at room temperature were prepared by a pHS-3C acidometer.

### 4.2. Analytical Procedures for Spectroscopic Experiments

The nitrate, chloride, or perchlorate salt of various cationic ions were processed into solution by dissolving in ultrapure water with concentration of 10 mM. The stock solution of **BHMMP** was prepared, according to this procedure, through dissolving **BHMMP** in pure ethanol and then diluting to 0.01 mM, in which 40% of the component was ethanol solvent. For spectrum measurement, a certain amount of metal ions was precisely added to the **BHMMP** solution with a pipetting gun, and the spectrum of the test solution, after uniform mixing, was recorded by UV–Vis and fluorescence spectrometers. In the fluorescence experiment, the excitation wavelength of the test solution was 370 nm, and the excitation, as well as emission slit widths, were all fixed to 10 nm.

### 4.3. DFT Investigation

In this paper, all DFT calculations were performed by the Dmol3 module of Materials Studio [65]. The Perdew–Burke–Ernzerholf (PBE) version of generalized gradient approximation (GGA) was utilized to treat the exchange correlation interaction [66]. To describe the long-range weak interactions, such as van der Waals force, the Grimme dispersion correction was used [67], and a double numerical basis set with polarization functions (DNP+) was applied for this system. The tolerance of the self-consistent field (SCF) was set as 1.0 × 10^−6^ Ha. During geometric optimizations, the convergence threshold parameters were 1.0 × 10^−5^ Ha (energy), 0.002 Ha/Å (maximum force), and 0.005 Å (maximum displacement), respectively.

### 4.4. In Vitro Cytotoxicity Assays

Human stromal cells (HSC) were plated at 1 × 10^5^ cells per well in a 96-well cell-culture plate, followed by incubation at 37 °C for 24 h. Then, the cells were incubated with varying concentrations of probes (0, 5, 10, 20, 30, 40, and 50 μM) for 24 h and washed with 100 μL fresh medium. Then, the fresh medium (100 μL) and MTT (10 μL, 5 mg/mL) were added to each well, and the cells were incubated for another 4 h at 37 °C. Finally, the absorbance of 560 nm was measured with a Bio-Rad microplate reader, and the cell viability was calculated (Figure S7).

### 4.5. Cell Imaging in HSC Cells

Following the reported method [68], cell imaging experiments were carried out using human stromal cells (HSC) that were exposed to DMEM/F-12 (1:1) medium at 37 °C in an atmosphere containing 5% CO_2_. Heat-inactivated FBS, penicillin, streptomycin, and sodium pyruvate are further supplemented to this medium in an appropriate amount to suit mammalian cell culture under low serum content. HSC were cultured in 6-well plates for 24 h, treated with Al^3+^ (0 and 50 μM) for 2 h, washed with Hanks’ balanced salt solution three times, and then seeded with **BHMMP** (10 μM) for 2 h. Finally, cell imaging was captured through a fluorescence microscope.

### 4.6. Synthesis of BHMMP

According to the reported literature, 5-(benzo[d]thiazol-2-yl)-2-hydroxy-3-methoxybenzaldehyde (**BHM**) was obtained via a condensation reaction between 2-aminothiophenol and vanillin, which was further used in the formylation through the Duff reaction [69].

The classic Schiff base reaction was shown in Scheme 1. Compounds **BHM** (100 mg, 0.35 mmol) and 2-aminophenol (38 mg, 0.35 mmol) were placed in a round bottom flask containing 15 mL ethanol, followed by adding two drops of acetic acid as a catalyst. The mixture was stirred for 8 h at room temperature, until the reactant was consumed. The precipitate was collected by filtration and washed by ethanol three times, and the desired product **BHMMP** (103 mg) was obtained after drying. Yield: 78 %. m.p. 250.9–251.7; ^1^H NMR (Figure S8) (600 MHz, DMSO-*d*_6_) δ (ppm) 15.24 (s, 1H), 10.23 (s, 1H), 9.23 (s, 1H), 8.11 (d, J = 7.8 Hz, 1H), 8.01 (d, J=8.0 Hz, 1H), 7.92 (s, 1H), 7.64 (s, 1H), 7.59 (d, J = 7.7 Hz, 1H), 7.52 (t, J = 7.5 Hz, 1H), 7.42 (t, J = 7.4 Hz, 1H), 7.18 (t, J = 7.5 Hz, 1H), 7.02 (d, J = 7.9 Hz, 1H), 6.95 (t, J = 7.5 Hz, 1H), and 3.93 (s, 3H); ^13^C NMR (Figure S9) (151 MHz, DMSO-*d*_6_) δ (ppm) 167.62, 161.98, 159.59, 154.10, 150.75, 134.60, 131.36, 128.89, 126.95, 125.42, 125.02, 122.71, 122.61, 121.45, 120.24, 119.95, 119.03, 117.71, 116.97, 111.67, and 56.24; HRMS: *m*/*z* (TOF MS ES^−^) (Figure S10), calcd. for C_21_H_16_N_2_O_3_S: 375.0803 [**BHMMP**-H^+^]^−^, found: 375.0808.

## 5. Conclusions

In summary, a Schiff-based probe **BHMMP**, revealed recognition towards Al^3+^, with an obvious optical color change, as well as a fluorescence-enhanced behavior in the EtOH/H_2_O (2/3, *v*/*v*, 0.01 M HEPES, pH = 5) solution. The detection limit was obtained from fluorescence titration data as 0.70 µM. The binding mode and working mechanism between the probe **BHMMP** and Al^3+^ was inferred by various spectra, HRMS, and ^1^H NMR titration. The 1:1 complex, formed between Al^3+^ and O/N atoms in **BHMMP**, inhibited the C=N isomerization and PET processes, thus opening fluorescent response. Importantly, not only could **BHMMP** be suitable for quantitatively monitoring Al^3+^ in real water samples and test paper, but it was also successfully applied for turn-on fluorescently sensing Al^3+^ in HSC cells. This work will provide an effective sensor for detecting Al^3+^ both environmentally and biologically.

## Data Availability

The data are available from the corresponding author on reasonable request.

## Supplementary Materials

The following supporting information can be downloaded at https://www.mdpi.com/article/10.3390/molecules27082569/s1. Figure S1. Fluorescence intensity probe BHMMP with varying concentration of Al^3+^. Figure S2. The absorbance of probe BHMMP with varying concentration of A^l3+^. Figure S3. Benesi–Hildebrand plot from fluorescence titration data of BHMMP (10 µM) with Al^3+^. Figure S4. Tauc plot of BHMMP and BHMMP–Al^3+^. Figure S5. Reversibility of BHMMP for Al^3+^ Figure S6. Fluorescent response of probe BHMMP in actual water samples. Figure S7. The cell viability of probe BHMMP. Figure S8. ^1^H NMR spectrum of probe BHMMP. Figure S9. ^13^C NMR spectrum of probe BHMMP. Figure S10. ESI-MS spectrum of probe BHMMP in DMF. Table S1 Comparison of previously reported Al^3+^ probes with functional groups similar to BHMMP. Table S2 The fluorimetric determination results for Al^3+^ in actual water samples by probe BHMMP.

## Sample Availability

Samples of the compounds are available from the authors.
