# Supplementary material for: A Significant Fluorescence Turn-On Probe for the Recognition of Al3+ and Its Application
Source: Molecules. 2022 Apr 15;27(8):2569. doi: 10.3390/molecules27082569 (PMC9028138; doi:10.3390/molecules27082569)
Supplement: Supplementary file 1 [file molecules-27-02569-s001.zip › molecules-1613811-supplementary.pdf]

Supplementary Materials

A Significant Fluorescence Turn-On Probe for the Recognition of Al^{3+} and Its Application

Zhiyong Xing [1,*], Junli Wang [2,3,*], Junhui Huang [4], Xiangfeng Chen [1], Ziao Zong [1], Chuanbin Fan [1] and Guimei Huang [1]

[1] School of Laboratory Medicine, Youjiang Medical University for Nationalities, Baise, Guangxi 533000, China; 01327@ymun.edu.cn (X.C.); zongziao@ymun.edu.cn (Z.Z.); 01363@ymun.edu.cn (C.F.); 00966@ymun.edu.cn (G.H.)

[2] Department of Reproductive Medicine, Affiliated Hospital of Youjiang Medical University for Nationalities, Baise, Guangxi 533000, China

[3] Environmental Health Risk Assessment and Prevention Engineering Center of Ecological Aluminum

Industry Base, Youjiang Medical University for Nationalities, Baise, Guangxi 533000, China

[4] Institute of Science and Technology Information, Baise, Guangxi 533000, China; 00497@ymun.edu.cn

* Correspondence: zyxing@ymun.edu.cn (Z.X.); baisewangjunli@ymun.edu.cn (J.W.)

Table of Contents

Figure S1. Fluorescence intensity at 522 nm of probe **BHMMP** (10 μM) with varying concentration of Al^{3+} (0-10 μM) in EtOH/H_2O (2/3, v/v, 0.01 M HEPES, pH = 5) medium.

Figure S2. The absorbance of probe **BHMMP** (10 μM) with varying concentration of Al^{3+} (0-10 μM) in EtOH/H_2O (2/3, v/v, 0.01 M HEPES, pH = 5) medium.

Figure S3. Benesi–Hildebrand plot from fluorescence titration data of **BHMMP** (10 μM) with Al^{3+} in EtOH/H_2O (2/3, v/v, 0.01 M HEPES, pH = 5) medium.

Figure S4. Tauc plot of **BHMMP** and **BHMMP**-Al^{3+} based on their corresponding absorption spectra.

Figure S5. Reversibility of **BHMMP** for Al^{3+}

Figure S6. Fluorescent response of probe **BHMMP** in actual water samples in EtOH/H_2O (2/3, v/v, 0.01 M HEPES, pH = 5) solution upon addition of different concentration of Al^{3+} (1, 3, 5, 7, 9 μM) at 522 nm.

Figure S7. The cell viability of probe **BHMMP**.

Figure S8. ^1H NMR spectrum of probe **BHMMP** in DMSO-d_6.

Figure S9. ^{13}C NMR spectrum of probe **BHMMP** in DMSO-d_6.

Figure S10. ESI-MS spectrum of probe **BHMMP** in DMF.

Table S1 Comparison of previously reported Al^{3+} probes with functional groups similar to **BHMMP.**

Table S2 The fluorimetric determination results for Al^{3+} in actual water samples by probe **BHMMP**.

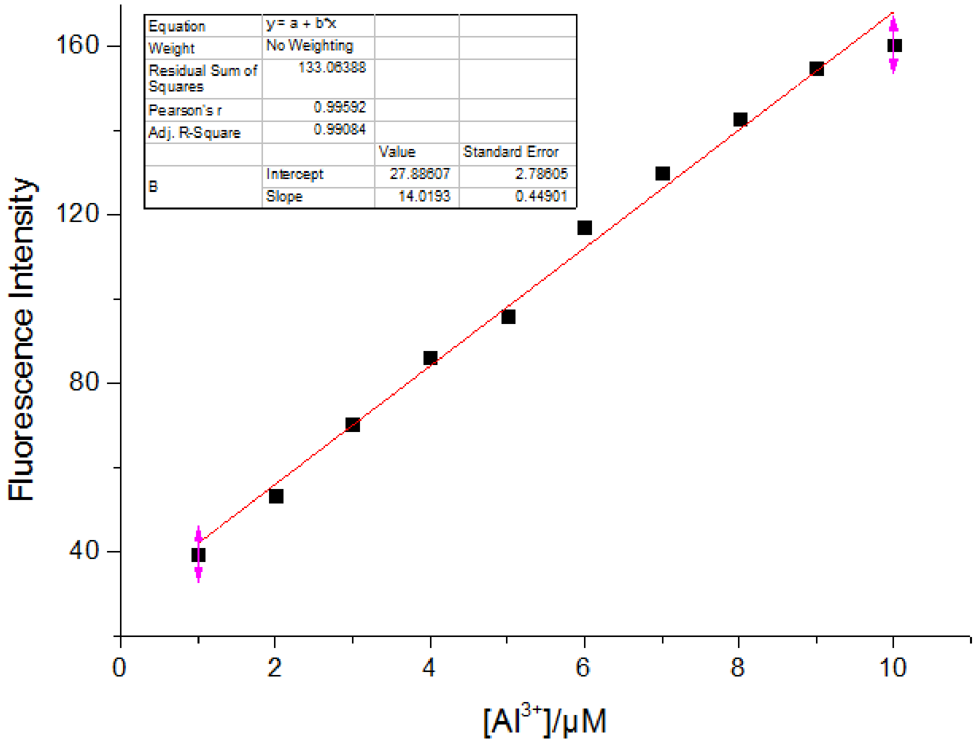

Figure S1. Fluorescence intensity at 522 nm of probe **BHMMP** (10 μM) with varying concentration of Al^{3+} (0-10 μM) in EtOH/H_2O (2/3, v/v, 0.01 M HEPES, pH = 5) medium.

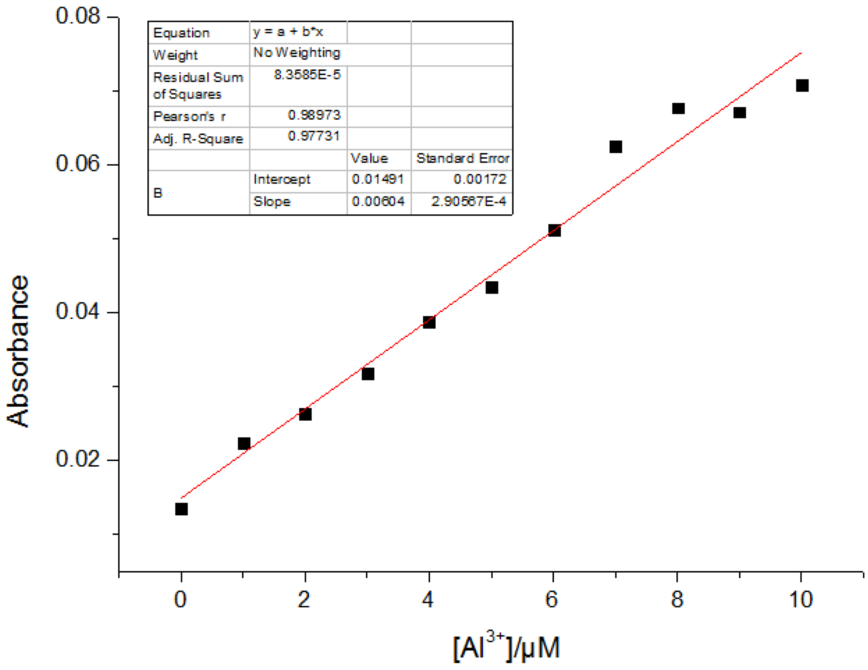

Figure S2. The absorbance of probe **BHMMP** (10 μM) with varying concentration of Al^{3+} (0-10 μM) in EtOH/H_2O (2/3, v/v, 0.01 M HEPES, pH = 5) medium.

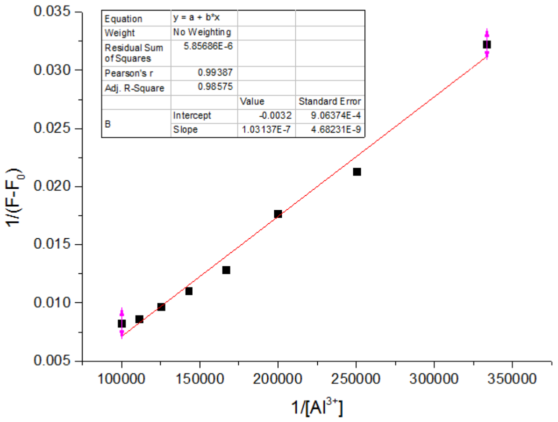

Figure S3. Benesi–Hildebrand plot from fluorescence titration data of **BHMMP** (10 μM) with Al^{3+} in EtOH/H_2O (2/3, v/v, 0.01 M HEPES, pH = 5) medium.

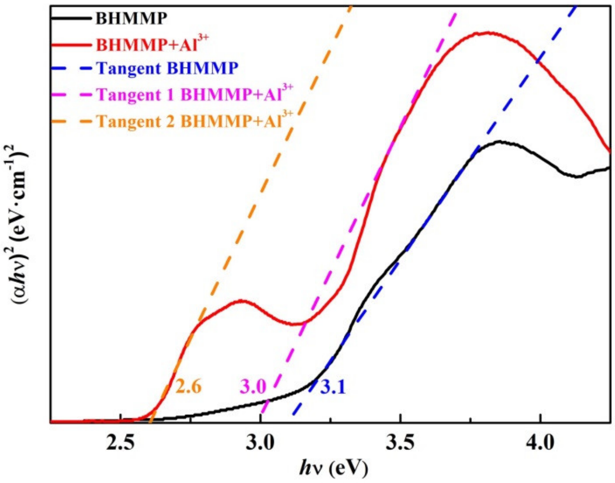

Figure S4. Tauc plot of **BHMMP** (black) and **BHMMP**+Al^{3+} (red) based on their corresponding absorption spectra.

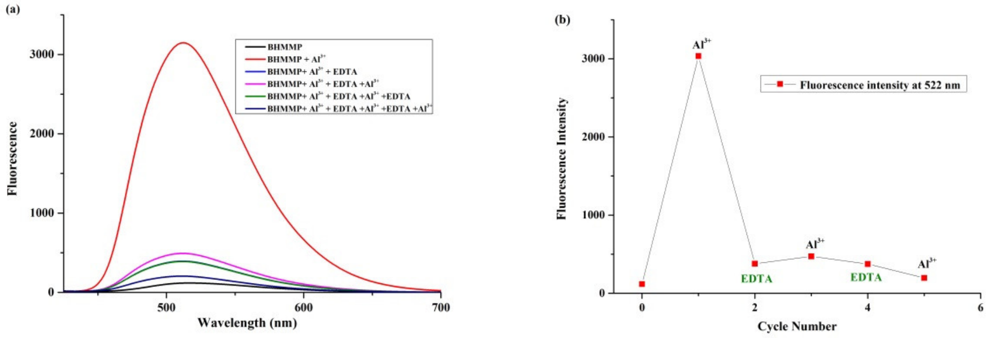

Figure S5. Reversibility of **BHMMP** for Al^{3+}: Fluorescence spectrum of **BHMMP** upon the alternative addition of Al^{3+} and EDTA (a); Fluorescence intensity at 522 nm upon alternative addition of Al^{3+} and EDTA.

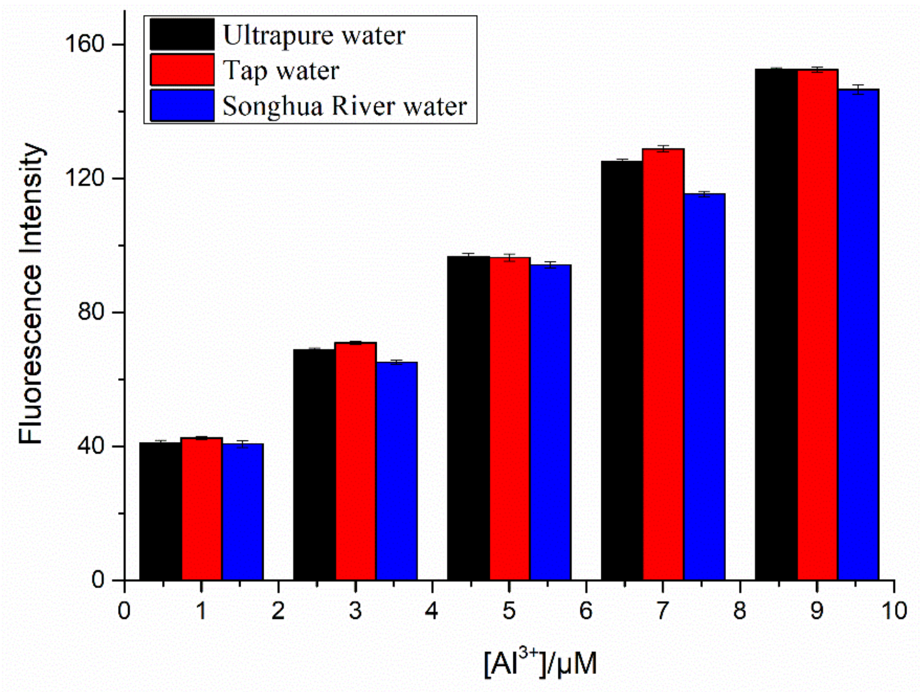

Figure S6. Fluorescent response of probe **BHMMP** in actual water samples in EtOH/H_2O (2/3, v/v, 0.01 M HEPES, pH = 5) solution upon addition of different concentration of Al^{3+} (1, 3, 5, 7, 9 μM) at 522 nm.

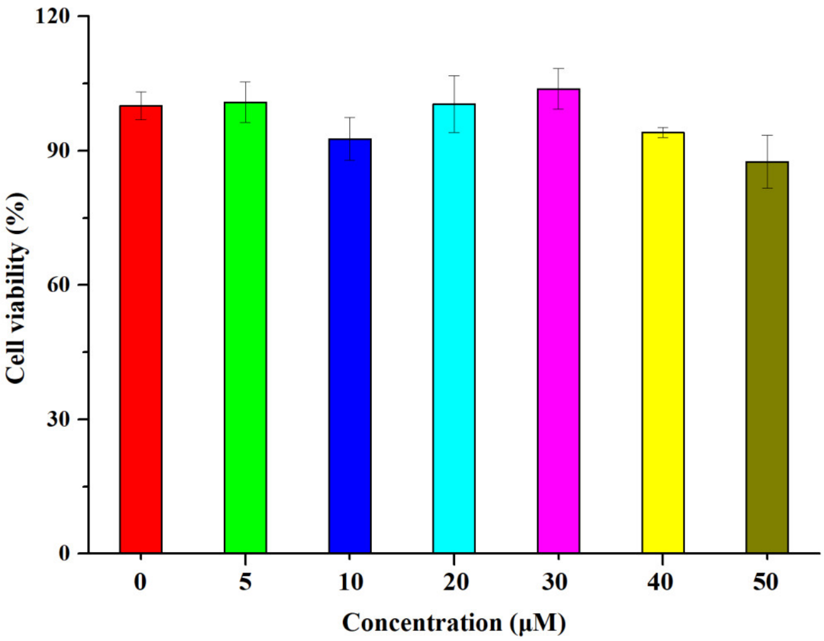

Figure S7. Cell viability of probe **BHMMP**

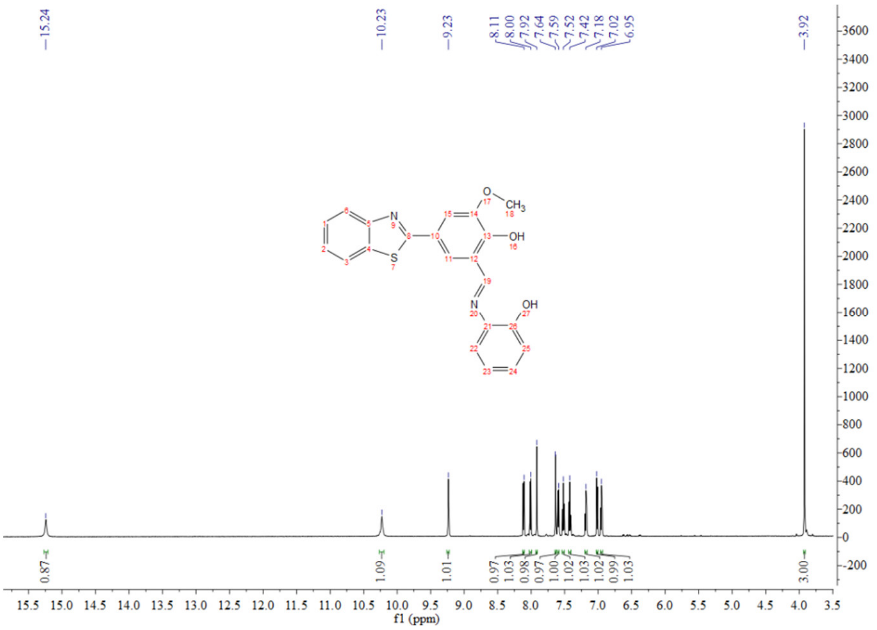

Figure S8. ^{1}H NMR spectrum of probe **BHMMP** in DMSO-d_6.

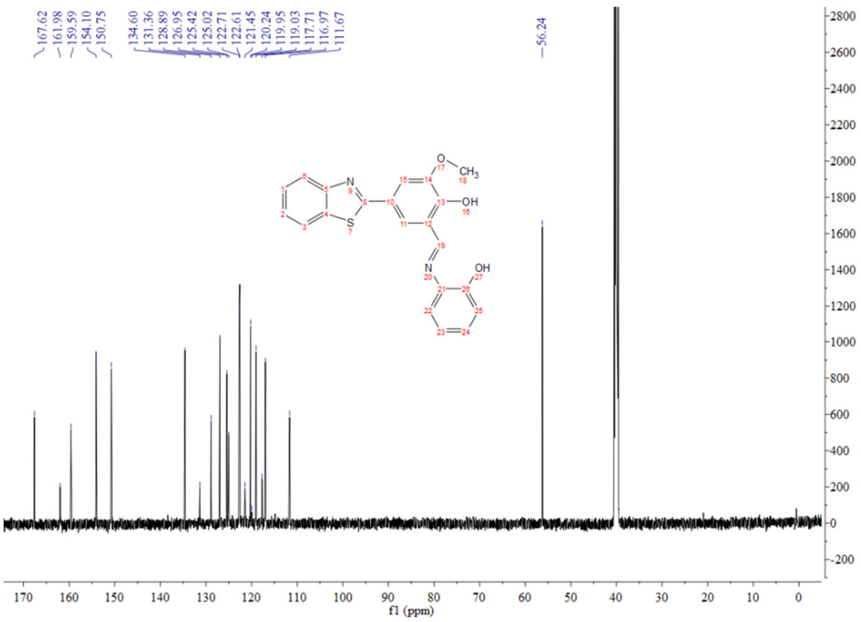

Figure S9. ^{13}C NMR spectrum of probe **BHMMP** in DMSO-d_6.

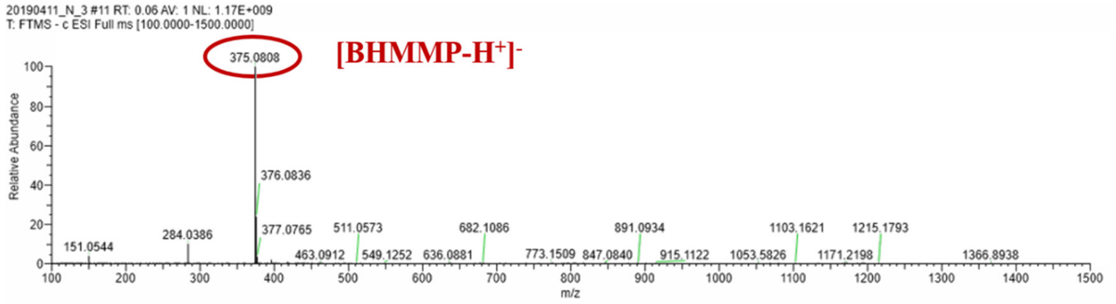

Figure S10. ESI-MS spectrum of probe **BHMMP** in DMF.

Table S1. Comparison of previously reported Al^{3+} probes with functional groups similar to **BHMMP.**

Ref.	Probes	Detection Medium	Fluorescence enhancement	LOD	Binding Constants	Quantum yield of complex	Maximum emission wavelength	Application
[46]	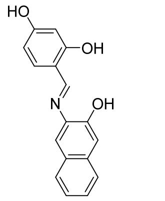	DMF/H_2O (9:1)	No report	0.49 μM	2.75×10^{3} M^{-1}	0.2508	390 nm	Test strips
[47]	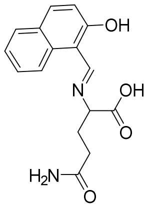	MeCN/H_2O (1:1)	6-fold	0.10 μM	No report	0.3402	433 nm	Cell imaging
[48]	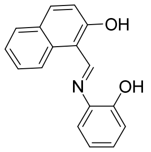	MeOH/H_2O (1:1)	7-fold	10.00 μM	3.40×10^{4} M^{-1}	No report	517 nm	Test strips<br>Cell imaging
[49]	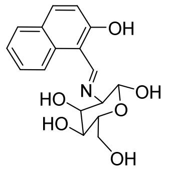	EtOH	60-fold	4.08 nM	5.75×10^{3} M^{-1}	0.71	427 nm	Cell imaging
50]	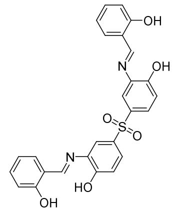	DMSO/H_2O (9:1)	52-fold	0.22 μM	1.71×10^{4} M^{-1}	0.198	485 nm	Water sample
[51]	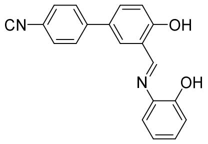	MeCN/H_2O (1:1)	24-fold	1.37 μM	5.36×10^{9} M^{-2}	0.1774	516 nm	No report
[52]	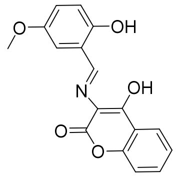	EtOH/H_2O (9:1)	7-fold	1.62 μM	9.90×10^{3} M^{-1}	No report	510 nm	Cell imaging
This work	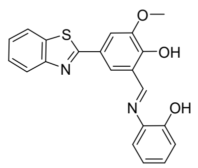	EtOH/H_2O (2:3)	38-fold	0.70 μM	3.10×10^{4} M^{-1}	0.11	522 nm	Water sample<br>Test strips<br>Cell imaging

Table S2. The fluorimetric determination results for Al^{3+} in actual water samples by probe **BHMMP**.

Water samples	Spiked amount (μmol/L)	Found amount (n=3) (μmol/L)	Recovery (n=3) (%)	RSD (%)	Relative error (%)
Ultrapure water	1	0.94	97.99	0.87	-2.01
	3	2.92	98.38	0.54	-1.62
	5	4.91	98.66	0.32	-1.34
	7	6.93	99.27	1.11	-0.73
	9	8.89	98.98	0.39	-1.02
Tap water	1	1.05	101.55	1.94	1.55
	3	3.07	101.40	1.79	1.40
	5	4.89	98.36	0.74	-1.64
	7	7.20	102.23	0.34	2.23
	9	8.89	98.97	0.56	-1.03
Songhua River water	1	0.92	101.83	1.11	1.83
	3	2.65	98.33	2.74	-1.67
	5	4.73	101.96	0.15	1.96
	7	6.24	97.30	1.78	-2.70
	9	8.47	101.23	0.41	1.23
